# A Comprehensive Mechanical Characterization of Subject-Specific 3D Printed Scaffolds Mimicking Trabecular Bone Architecture Biomechanics

**DOI:** 10.3390/life13112141

**Published:** 2023-10-31

**Authors:** Laura Rojas-Rojas, Gianluca Tozzi, Teodolito Guillén-Girón

**Affiliations:** 1 Materials Science and Engineering School, Tecnológico de Costa Rica, Cartago 30109, Costa Rica; tguillen@itcr.ac.cr; 2 School of Engineering, University of Greenwich, Chatham ME4 4TB, UK; g.tozzi@greenwich.ac.uk; 3 School of Mechanical and Design Engineering, University of Portsmouth, Portsmouth PO1 3DJ, UK

**Keywords:** polymeric scaffold, trabecular bone, mechanical properties, microCT, digital volume correlation

## Abstract

**Simple Summary:**

This study aimed at producing a polymeric scaffold with the ability to replicate the structure and mechanical properties of native trabecular bone. The scaffold’s morphological characteristics and mechanical parameters were compared to those of trabecular bone from bovine iliac crest. Using a three-step procedure, the elastic modulus and yield strength were investigated, and a dynamic test was performed to evaluate the mechanical behavior under various loading regimes. The local micromechanics of the scaffolds were assessed with in situ microcomputed tomography and digital volume correlation, which measured the full-field strain distribution. Overall, the results showed that the fabricated polymeric scaffold exhibited mechanical properties in the range of trabecular bone and represents a suitable bone surrogate for in vitro applications, with the potential to be further translated for in vivo clinical purposes.

**Abstract:**

This study presents a polymeric scaffold designed and manufactured to mimic the structure and mechanical compressive characteristics of trabecular bone. The morphological parameters and mechanical behavior of the scaffold were studied and compared with trabecular bone from bovine iliac crest. Its mechanical properties, such as modulus of elasticity and yield strength, were studied under a three-step monotonic compressive test. Results showed that the elastic modulus of the scaffold was 329 MPa, and the one for trabecular bone reached 336 MPa. A stepwise dynamic compressive test was used to assess the behavior of samples under various loading regimes. With microcomputed tomography (µCT), a three-dimensional reconstruction of the samples was obtained, and their porosity was estimated as 80% for the polymeric scaffold and 88% for trabecular bone. The full-field strain distribution of the samples was measured using in situ µCT mechanics and digital volume correlation (DVC). This provided information on the local microdeformation mechanism of the scaffolds when compared to that of the tissue. The comprehensive results illustrate the potential of the fabricated scaffolds as biomechanical templates for in vitro studies. Furthermore, there is potential for extending this structure and fabrication methodology to incorporate suitable biocompatible materials for both in vitro and in vivo clinical applications.

## 1. Introduction

Bone is a heterogeneous, anisotropic, and load-bearing mineralized tissue. Its structure exhibits a complex distribution of trabeculae, laminae, and pores, influenced by mechanical loadings, skeletal homeostasis, and the organism’s phosphocalcic balance [1,2]. Collectively, these factors confer both strength and stiffness to the structure while also accommodating organic components, including blood vessels, marrow, and nerves. [3,4]. Bone mass can diminish due to factors such as aging, degradation resulting from estrogen or androgen deficiency, among other influences, leading to a reduction in both density and mineral composition. When this happens, a number of human conditions, such as osteoporosis, emerge, and people become more vulnerable to trauma accidents and illness [5]. Bone can also be injured, producing large defects that cannot heal spontaneously, and the management of critical-sized bone defects remains a major clinical need, requiring novel biomaterials and treatment procedures [6]. Tissue engineering scaffolds represent a viable solution to aid the repair and functional restoration of bone [7,8], thus, considerable effort has been put into the fabrication of scaffolds that mimic trabecular bone structure. Cell-seeded 3D scaffolds may be used as in vitro study models in drug studies or to assess cell growth in bone remodeling [9,10]. 

Porous polymeric scaffolds have demonstrated their success for in vitro applications [9], generating bone-like structures that closely replicate the properties of real bone. Such structures are versatile and incorporate a wide range of applications [11]. Porous scaffolds can be designed in solid-free form or by a reverse engineering approach [12,13,14]. The solid-free form includes printing a periodic structure or lattice by fused deposition modeling (FDM) square and hexagon patterns [15,16] or by foams or gas injection in hexagonal structures [17]. In contrast, reverse engineering is based on reproducing or printing a 3D reconstruction of a biological material. The fabricated structure is aleatory and presents a replica of the bone [12]. Scaffold fabrication techniques encompass a range of methods, including additive manufacturing (AM), stereolithography (SLA), laser sintering, powder sintering, and various other techniques [18,19,20]. Scaffold materials vary significantly depending on their intended applications. For instance, structural materials are employed in load-bearing applications, while different functional materials are used for healing purposes [21]. Among the functional materials are hydrogels, natural polymers, composites, as well as synthetic and natural polymer [7,20]. Various materials and fabrication techniques for bone tissue engineering are found in the literature [7,21,22,23]. 

Various types of photosensitive polymeric materials or resins are currently employed in the fabrication of scaffolds through SLA [24]. However, SLA resins are not inherently biocompatible. Therefore, scaffolds produced with this technique must undergo either surface treatment or bulk modification to meet the biocompatibility requirements for cell adhesion, colonization, and subsequent implantation. In addition to biocompatibility, scaffolds must address other functionalities like osteoconductivity and osseointegration. These factors are crucial when considering 3D scaffolds as implants meant to be used as proliferation sites to enhance and guide cell growth. Osseointegration refers to the chemical bonding of the interface between the scaffold and its surrounding native bone [18,21,25,26]. Chang et al. [27] provide insights on several important aspects of osseointegration.

SLA-polymeric resins can be chemically modified to benefit biocompatibility and osseointegration [28] by incorporating fillers or additives [19,20,29]. The most common inorganic additives include tricalcium phosphate (TCP) and hydroxyapatite (HAp), while organic additives like collagen and gelatin are also used [8,18]. An alternative approach is to apply biological reaction layers or surface coatings on the scaffold’s surface [29]. Different surface treatment methods have been presented by Yang et al. [14]. For instance, they discuss the use of a direct coating method achieved through physical vapor deposition or chemical vapor deposition [30].

In the process of fabricating bone scaffolds, it is important to consider the printer’s resolution, printer speed, and manufacturing costs, as indicated by relevant research [8]. While manufacturing 3D scaffolds for bone tissue engineering, an equilibrium between these factors is needed, which may result in a compromise of the scaffold’s resolution or porosity, particularly if the adhesion of surface coatings significantly augments biocompatibility. Furthermore, pertinent to implant-related scaffolds, factors such as geometric shape, mechanical and architectural properties must be meticulously factored into the design and manufacturing process [28]. 

Mechanical properties are crucial for bone substitutes, and the focus must be on matching or mimicking the biomechanical requirements [20]. These requirements encompass features, such as pore connectivity and mechanical behavior, ensuring the scaffold can faithfully replicate the in vivo functionality of the tissue [17,31,32]. Ultimately, the mechanical properties of trabecular bone and its biological properties are related because both depend on its architecture, pore size, and distribution [3,19,33] with adequate structural support for physiological loading [34,35]

Traditionally, the mechanical behavior of porous scaffolds is assessed by uniaxial/cyclic compression [36,37,38]. However, understanding the local mechanics of porous biomaterials (i.e., local strain distribution) is very important to evaluate the effect of microdamage on their overall mechanical competence. Digital volume correlation (DVC) based on in-situ X-ray microcomputed tomography (µCT) is a powerful tool with the ability to measure the three-dimensional (3D) full-field strain in bones, biomaterials, and bone-biomaterial systems, as recently reviewed by Dall’Ara and Tozzi [39]. This study made use of SLA-3D printing to produce a bone-like scaffold via a reverse engineering approach. The main goal was to obtain a scaffold with similar mechanical and morphological properties as native trabecular bone. The scaffold was fabricated and characterized. Its mechanical properties (i.e., E, σ_y_, and σ_ult_) were estimated and compared to those of native trabecular tissue. The full-field strain distribution of scaffolds and bone was measured via DVC from in situ µCT. In addition, to study the dynamic mechanical response of the scaffolds, a step fatigue test was performed.

## 2. Materials and Methods

### 2.1. Sample Preparation

#### 2.1.1. Trabecular Bone Sample Preparation

Trabecular bone samples were harvested from fresh bovine iliac crest. An 8 mm milling drill was used to extract cylindrical-shaped samples of 12 mm length. After collecting the samples, they were wrapped in PBS-soaked gauze and stored at −4 °C. The samples were imaged with a Versa 520 X-ray microscope (Carl Zeiss Ltd., Pleasanton, CA, USA) operating at 1.11 kV, 87 µA 1016 projections over 360° at 2 s exposure time to achieve 15 µm voxel size after reconstruction. The 3D reconstruction of the standard triangle language (STL) trabecular model was obtained using VGSTUDIO MAX (VGStudio MAX 2.0, Volume Graphics, Germany) software. The model was upscaled 1.4 times to fit the printer’s resolution. 

#### 2.1.2. Polymer Sample Preparation

Polymeric samples were fabricated using a Form 3B+ SLA printer, FormLabs^®^, with a 100 µm maximum printing resolution. Dental LT^®^ (FormLabs) was chosen as the resin for model printing. In this work, Dental LT is also referred to as SLA resin. After printing, the samples were cleaned with Isopropyl alcohol (IPA) in an ultrasonic bath for 3 min. Uncured material was removed from the inside of the cylindrical-porous structure by pressurized air. The samples were placed in IPA in an ultrasonic bath for 3 min. Then, the fabricated porous samples were cured with UV light, λ = 405 nm, at 60 °C for one hour according to the manufacturer’s instructions. Samples were exposed to UV light using a FormCure^®^. The printing supports were removed and discarded. Samples were then stored in a controlled environment, 22 °C and 35% humidity until they were used. The dimensions of the printed SLA cylindrical-porous sample were 18 mm in length and 11 mm in diameter. 

### 2.2. Sample Characterization

The surface was imaged using scanning electron microscopy (SEM), HITACHI TM-3000, with an acceleration voltage of 15 kV and an emission current of 45 mA. The internal morphology of the samples was analyzed by μCT, using the same system settings reported above. Image analysis and morphometric parameters were calculated using Fiji [40,41]. The measurement of these parameters was performed by selecting a uniform threshold with a circular region of interest (ROI). The scaffold’s surface was excluded from the ROI to remove the superficial debris due to sample preparation. The selected ROI´s diameter was 7 mm for trabecular bone and 10 mm bone scaffold, respectively. BV/TV, Tb.Th and Tb.Sp were estimated using this ROI.

### 2.3. Mechanical Characterization

For the mechanical evaluation of the samples, a servohydraulic test system MTS Bionix Tabletop Model 370.02 (Axial/torsional) with a 2500 N load cell was used. End caps were attached to the samples for mechanical testing to minimize end artifacts and to guarantee cross-sectional loading, reducing shear stress [42,43]. To estimate the elastic and dynamic properties of the samples, several tests were conducted. Monotonic compressive tests, in situ µCT mechanics, and dynamics tests were conducted on the samples. The details of the methodology are explained in the following sections. 

#### 2.3.1. Monotonic Compressive Tests on Trabecular Bone

Six trabecular bone sample was monotonically compressed under displacement control at a deformation rate of 0.028 mm/s and a preload of −15 N following ASTM D1621-16 (2016) [44]; ASTM D695-15 (2015) [45]. Mechanical parameters were reported as the average for the six measured samples. The experimental yield strength (σ_mB_) was calculated as 0.2% off-set. Then, the yield stress at 70% (σ_70%_) and the yield stress at 40% (σ_40%_) were estimated as σ_70%_ = 0.7σ_mB_ and σ_40%_ = 0.4σ_mB_. These parameters were used to set up the three-step procedure described by Krupp et al. [46] and ISO 13314:2011 (2011) [47]. 

Six samples were used for the three-step procedure. This methodology consisted of an initial compression at a constant strain rate of 0.0028 mm/s until σ_70%_ was reached, then unloading of the sample at 2.8 mm/s to σ_40%_, and then, loading of the sample at a strain rate of 0.028 mm/s until strain reached ε = 20%. The mechanical parameters of trabecular bone were calculated from the average of the samples. E was calculated as the slope of the unloading section. σ_y_ was obtained as the 0.2% off-set, and σ_ult_ was obtained as the maximum value before a decrease in stress after yielding of the sample.

#### 2.3.2. Monotonic Compressive Tests on SLA Scaffolds

Six SLA samples followed the same methodology as described for trabecular bone. They were monotonically compressed under displacement control at a deformation rate of 0.028 mm/s and a preload of −15 N, then the experimental yield strength (σ_mS_) was calculated as the 0.2% off-set. The yield stress at 70% (σ_70%_) and the yield stress at 20% (σ_20%_) were estimated as σ_70%_ = 0.7σ_mS_ and σ_20%_ = 0.2σ_mS_. These parameters were calculated as the average for the six measured samples.

Then, the three-step procedure was set up using these parameters. An initial compression was set at a constant strain rate of 0.0028 mm/s until σ_70%_ was reached, then unloading of the sample at 2.8 mm/s back to σ_20%_ and then, loading of the sample at a strain rate of 0.028 mm/s until strain reached ε = 20%. The mechanical parameters, ultimate stress (σ_ult_), compressive yield stress (σ_y_), and E, were calculated as previously explained for trabecular bone. 

### 2.4. In Situ Mechanics and Digital Volume Correlation 

The micro-deformation study was performed using in situ stepwise µCT (Carl Zeiss Ltd., Pleasanton, CA, USA) mechanics with a 5 kN load cell (CT5000, Deben Ltd., Bury Saint Edmunds, UK). Three strain steps were selected for image acquisition; the first compressive strain (ε_st1_) was selected within the elastic region of the sample where σ_20%_ < σ_1_ < σ_70%_. The second compressive strain (ε_st2_) was selected near σ_y_. The third compressive strain (ε_st3_) was selected after the sample had failed. Acquisition was performed using the same µCT settings as explained in previous Section 2.1.1. 

Two sets of µCT scans were collected at the beginning of the test, without any loading for an estimation of the uncertainty [48]. The samples (3 for trabecular bone and 3 for SLA scaffolds) were then compressively loaded at 1 mm/s until ε_st1_ was reached and then loaded again up to ε_st2_ and ε_st3_ at the same compression rate. Following each compression step the sample was allowed to relax for 15 min before tomographic acquisition. 

Digital volume correlation (DVC) was used to measure the full-field strain distribution on both SLA and trabecular bone structures using DaVis software (v8.3, LaVision, Germany). DVC was performed between the first image and those at ε_st1_, ε_st2_, and ε_st3_ strain steps to compute the 3D full-field normal strain in the direction of loading (ε_zz_). A multi-pass scheme with a final sub-volume of 48 voxels (671 µm), reached via predictor passes using sub-volumes of 72, 64, and 56 voxels was used [49,50]. The strain visualization onto the reconstructed image was performed with Avizo 8.1 software (Thermo Scientific). With a 48 voxel size, the mean absolute error (MAER) and the standard deviation of the error (SDER) [51,52,53] of the strain components were found to be <600 and 173 µε, respectively, in all cases. 

### 2.5. Dynamic Compressive Tests

The dynamic compressive test was designed to assess the deformation behavior of the SLA samples as compared to that of native trabecular tissue. A long multi-step test, also referred to as stepwise dynamic behavior, was used for trabecular bone and SLA sample (*N* = 6). This procedure consisted in discretely increasing the compressive load on the sample every 5000 cycles [54]. The frequency was kept at a constant value of 2 Hz. The amplitude values (σ_min_ and σ_max_) were determined using σ resulting from the three-step procedure. σ_max_ was kept at 0.01σ_y_ and σ_min_ varied from 0.2σ_y_, 0.4σ_y_, 0.6σ_y_, and 0.8σ_y_. 

## 3. Results

### 3.1. Sample Characterization

**Figure 1:**
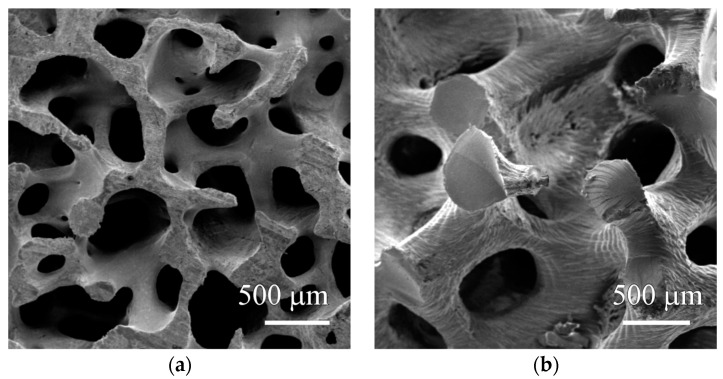
SEM micrographs for a cross-section of the (**a**) trabecular bone (88% of porosity) and (**b**) SLA 3D printed construct (70% of porosity).

Trabecular bone average porosity was 88%. Figure 1a shows the morphology of the bone sample with struts and pores. The μCT analysis of the iliac crest bone had Tb.Th of 177 ± 27 μm and Tb.Sp was 1132 ± 132 μm. The fabrication procedure of the SLA scaffold resulted in a cylindrical porous and aleatory structure that represented an upscaled model of a natural-shaped trabecular bone. Figure 1b shows how strut thickness was greater than 100 μm. The trabecular spacing depended on the appropriate cleaning of the scaffold; the SEM micrograph shows clear circular pores. The morphometric parameters were calculated for the samples with a BV/TV = 0.30 ± 0.06, indicating the porosity of the scaffold was 70%. The resulting Tb.Th was 724 ± 144 μm, and the Tb.Sp was 2078 ± 134 μm.

### 3.2. Mechanical Characterization of Trabecular Bone and Bone Scaffolds 

**Figure 2:**
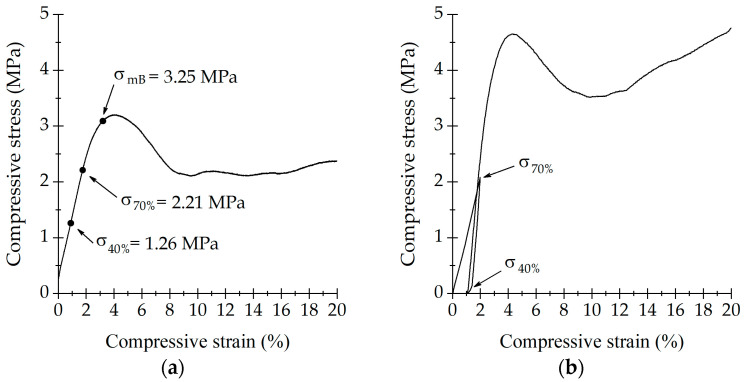
Compressive results for trabecular bone. (**a**) A monotonic compressive test and (**b**) the three-step procedure.

Figure 2 shows the results for monotonic compression and for the three-step procedure of trabecular bone. In Figure 2a, the monotonic compression of trabecular bone and the results for σ_mB_, σ_70%_ and σ_40%_. In Figure 2b, the three-step procedure of trabecular bone is shown. These results show an initial linear elastic behavior, up to 3% strain. Then it reached plasticity, and σ_ult_ was found at 4.5%. At 5% strain, there was a reduction in stress followed by a small slope after 10%. After ε = 12%, the cylindrical-porous structure collapsed, and densification started, this was evident as stress began to increase.

**Figure 3:**
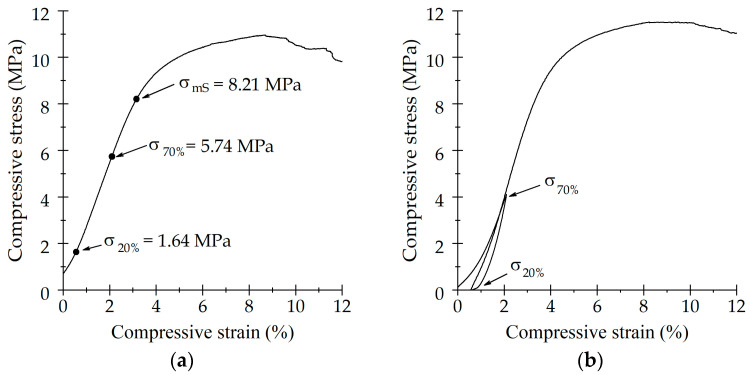
Compressive results for SLA scaffold. (**a**) A monotonic compressive test and (**b**) the three-step procedure.

Figure 3 shows the resulting σ_mS_, σ_70%_ and σ_20%_ for the monotonic compression and for the three-step procedure of SLA samples. The fabricated SLA-bone sample had a similar elastic behavior to that of the trabecular bone. SLA sample showed a linear behavior up to 4.5% strain. However, a greater plastic deformation was present for SLA (5% < ε < 11%).

The measured parameters for bone and SLA are reported in Table 1. Both samples had similar elastic moduli. However, σ_ult_ and σ_y_ were higher for the SLA sample.

### 3.3. Micromechanics and Digital Volume Correlation

**Figure 4:**
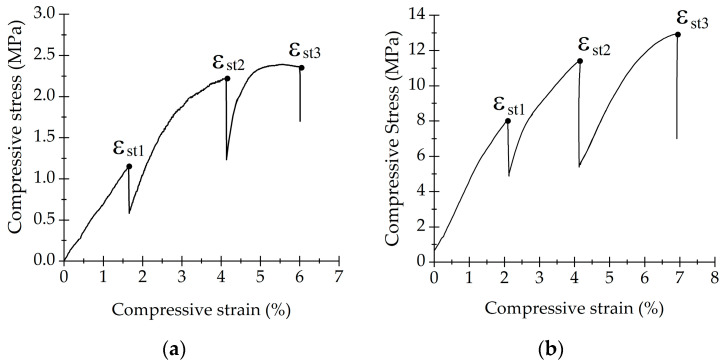
Stress-strain behavior of (**a**) trabecular bone and (**b**) SLA scaffold used for µCT analysis.

The goal of this test was to evaluate the full-field strain distribution in SLA when compared to trabecular at three different mechanical regimes: the elastic region, the plastic region, and post-yielding. Figure 4a,b shows the stepwise compressive stress as a function of the strain for bone and SLA samples, respectively. The steep reduction in stress corresponded to the relaxation time prior to each µCT acquisition.

**Figure 5:**
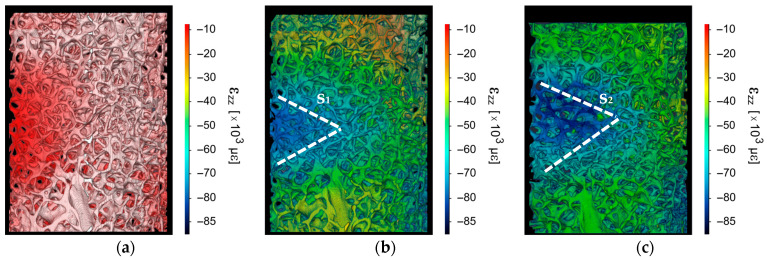
Bone reconstruction and strain distribution for (**a**) ε_st1_ = 1.6%, (**b**) ε_st2_ = 4%, region s_1_ depicts the section with higher strain concentration, and (**c**) ε_st3_ = 6%, region s_2_ depicts the section with higher strain concentration.

**Figure 6:**
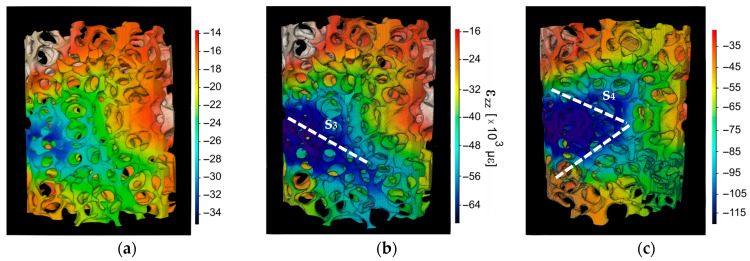
SLA scaffold reconstruction and strain distribution for (**a**) ε_st1_ = 2% (**b**) ε_st2_ = 4%, region s_3_ depicts the section with higher strain concentration, and (**c**) ε_st3_ = 6%, s_4_ presents the section with higher strain concentration.

DVC was used to quantify ε_zz_ (µε) distribution in both cellular materials. Figure 5 and Figure 6 show the strain maps rendered over the reconstructed µCT volume. Figure 5a shows the strain accumulation on trabecular bone during elastic deformation where ε_zz_ remained largely within −10,000 µε with peaks to ~−20,000 µε. The strain distribution (ε_zz_) during plastic deformation reached a maximum strain magnitude of ~−85,000 µε, noted by s_1_ (Figure 5b). This was located in the outer-middle section of the bone sample. As loading increased, the strain region grew larger, see Figure 5c. However, it remained in the middle of the bone sample s_2_.

Figure 6a shows the strain accumulation during elastic deformation of the SLA sample. The highest strain magnitude reached was ~−34,000 µε. Figure 6b shows the strain accumulation during plastic deformation, the strain concentrated as an inclined plane noted by s_3_ and had a value of ~−64,000 µε. Region s_4_ shows the strain concentration after the sample had yielded. It reached −115,000 µε, see Figure 6c. The variations on the ε_zz_ scale between Figure 6a–c corresponds to incremental strain proportions. This prompted us to maintain consistent color maps while adjusting the value ranges. 

For both specimens, the strain progression was similar. Maximum strain magnitude was concentrated mainly in the middle of the sample and progressed as an inclined plane. However, the measured strain was consistently higher for SLA sample than for trabecular bone. 

### 3.4. Dynamic Behavior 

**Figure 7:**
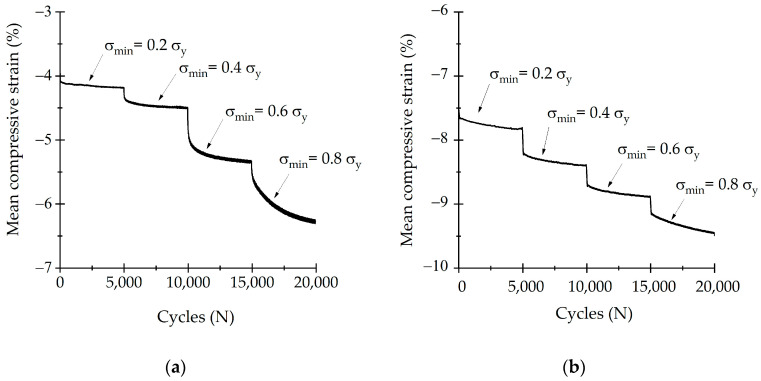
Step dynamic behavior of (**a**) trabecular bone and (**b**) SLA scaffold.

Samples were studied with the multi-step test to examine strain accumulation during 5000 cycles and strain variation with load increase. Mean stress was calculated as σ = │σ_max_ + σ_min_│$2^{-1}$. Figure 7a shows σ_mean_ as a function of cycle number (N) for bone. Strain increased with each compressive step every 5000 cycles. Mean strain varied slightly for the first three compressive steps where σ_min_ was 0.2σ_y_, 0.4σ_y_, 0.6σ_y_. However, for 0.8σ_y_, mean strain increased from 5.5% to 6% during the 5000 cycles. The multi-step test for SLA samples is depicted in Figure 7b. Compressive mean strain remained similar between each compressive step. For both samples, mean strain increased for every step according to the applied load. 

**Figure 8:**
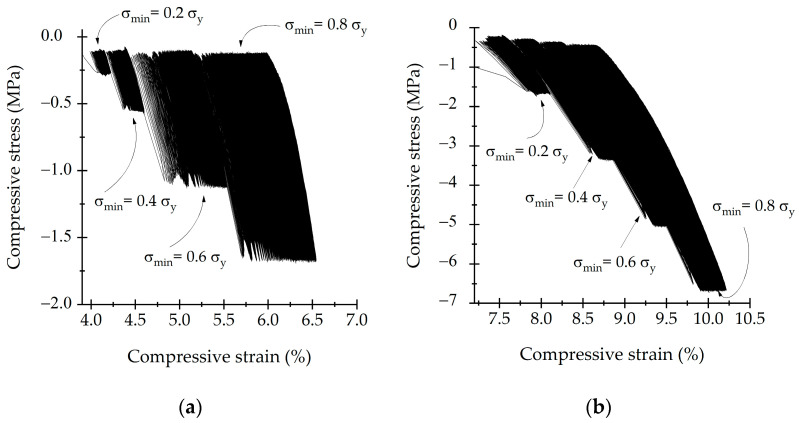
Hysteresis loops of (**a**) trabecular bone and (**b**) SLA scaffold in step loading conditions.

Hysteresis loops of the multi-step test are shown in Figure 8. The resulting hysteresis loops indicated no significant damage occurred on the samples for 5000 cycles. This was evident because the loops exhibited a slow shift in the strain axis. Successive hysteresis loops did not show broad changes in their slopes. Dynamic loading induces accumulative deformation on samples. However, there was no material damage when the load was increased.

## 4. Discussion

This study focused on the development of customized scaffolds designed to replicate the mechanical and morphological characteristics of trabecular bone. The biomechanical properties of the scaffolds were compared with those of analogous animal tissue to enhance the understanding of the macro- and micro-biomechanics associated with such scaffolds. 

The comparative analysis highlighted where the biomechanical performance of the scaffold could be improved. These results are important as they could have significant implications for scaffold design and fabrication as well as the ability to support osteointegration, which is intrinsically linked to the application of loads and structural orientation [25,27,28,55]. It is important to emphasize that this study represents the initial stage in assessing the feasibility of this structure as an implant. Subsequent research will encompass comprehensive in-vitro and in-vivo investigations, including biocompatibility.

SLA trabecular bone samples were fabricated and characterized, aiming to assess their suitability as scaffolds for biomechanical tissue engineering applications. Tissue engineering benefits from naturally shaped scaffolds to reproduce the native environment for cell growth [56,57]. This study focused on evaluating the mechanical performance of a 3D-printed scaffold under compressive loads and compared it to that of native trabecular bone. Therefore, special attention was given to the scaffold’s mechanical properties and architecture, as they needed to replicate the mechanical behavior of the anatomical bone they aimed to mimic [32,56]. Both structures, bone tissue, and SLA sample, were mechanically tested under monotonic and dynamic methods to assess their elastic behavior and examine their structural integrity in a dynamic test. In addition, SEM observation was performed on the samples to investigate pore connectivity and strut distribution. The average porosity of trabecular bone lies within what has been reported previously as 70% to 95% [58]. To determine whether SLA could replace bone mechanical function as a scaffold for bone-cell formation, the morphology and architectural organization of the SLA samples were evaluated by µCT [6]. Moreover, µCT analysis, also coupled with in situ mechanics and DVC, was performed to derive the morphometric parameters of both structures [6,59,60] and describe their microdeformation behavior [39].

Micrographs showed the printed structure resembled real bone as it had rod-like structures and pores (Figure 1). This characteristic was preserved by using the reverse modeling 3D printing methodology. This printing strategy preserved the native bone structure and this way the structure was duplicated [14,61]. This technique provided the advantage of creating a structure that directly mimicked the hierarchy, anisotropy, and porosity of native bone [12,62]. A biomimetic scaffold promotes cellular adhesion and stimulates the growth of bone [20,32]. High porosity found in SLA scaffolds was considered beneficial for the fabrication of bone tissue engineering scaffolds as it promotes cell proliferation and enhances permeability and extracellular matrix mineralization [20,32,35,36]. Onal et al. [63] reported that a pore size of 300 μm was required for adequate bone growth. Turnbull et al. [31] explained that pore size determined cell migration into scaffolds. The printed 3D SLA had a porosity within this range, which would make it suitable for in vitro cell growth experiments. The morphometric parameters of the SLA increased when compared to native bone tissue. Tb.Th for SLA was ~4 times higher than for bone. Tb.Sp was ~2 times higher.

The fabricated SLA sample showed promising mechanical properties with a mechanical response similar to native trabecular bone. The mechanical response of SLA was similar to the one for other cellular materials [62,64]. The main difference between SLA and the native bone stress behavior was found when they reached plasticity, for SLA, a broad plastic deformation was present, while for native bone plasticity was present for 3–6% and then followed by a plateau. The elasticity was 329 MPa for SLA and 336 MPa for bone. The elastic modulus for trabecular bone was reported in the range of 100 MPa to 20 GPa when going from apparent to tissue level [3], indicating a wide variation and heterogeneity in bone mechanical properties [33,65]. The increase in the SLA properties was attributed to the scaling of the model because scaling caused an increase in strut size. The struts are load-bearing structures and are responsible for force distribution in bone; therefore, their configuration and density determine the stiffness and strength of bone [3,66]. The mechanical parameters of the SLA scaffold were similar to those published in the literature, thus they have the potential to provide a suitable template for in vitro tissue engineering [67,68]. 

The resulting full-field strain magnitude of the SLA was higher than that measured for trabecular bone. The difference between them was attributed to the higher trabecular thickness found on the SLA samples due to fabrication. Strain accumulated in the middle section of the SLA samples for the three compressive steps: elastic, yielding, and post-yielding. In addition, the SLA sample and native bone showed similar strain accumulation profiles. The yield strain of bone at the tissue level in compression is between 8000 and 10,000 µε [69], when using DVC it is possible to appreciate regions under yield strain although compressed within the elastic apparent regime. Porous biomaterials typically experience higher local strains compared to bone, as reported by Bonithon et al [70]

Locating the strain accumulation areas was important to identify sites where higher mechanotransducive cell signals will concentrate [71], as also suggested by Pobloth et al. [72], who showed higher regenerative responses at the maximum principal strains in titanium bone scaffolds. 

The multi-step test was used to determine the loading stress range of the SLA structures. Samples could be loaded up to 0.8σ_y_ without losing their integrity, suggesting how SLA would be suitable for a longer dynamic. When abrupt changes in hysteresis loops are shown, these are indicative of material damage [37]. A longer dynamic test is required since in vitro cell growth has a duration of 1 to 3 days [73] but could last even for 21 consecutive days [1,74]. Since samples kept their integrity, these scaffolds can be used as a biomechanical scaffold for cell growth under mechanical stimulation loaded with σ_min_ < 0.8σ_y_.

Future studies will focus on the use of SLA samples as bone scaffolds. Specifically, we will investigate cell adhesion, distribution, and proliferation, building on the morphology and biomechanical properties of the SLA scaffold addressed in this study. Additionally, we intend to subject cell-inoculated scaffolds to various compressive loading patterns to explore cell differentiation outcomes and regeneration, leveraging the mechanical properties identified in this research. These steps are crucial, particularly in assessing compatibility with vascular tissue and angiogenesis-inducing properties.

## 5. Conclusions

The mechanical stability, geometrical features, and biocompatibility of porous structures are essential characteristics to obtain a functional bone tissue engineering scaffold. This study reported a simple fabrication method that produced a 3D highly porous polymeric scaffold. Its characterization proved how the fabricated structure was a reliable morphological model. The monotonic compressive properties of the SLA samples showed promising elastic properties that mimicked native bone. According to dynamic compressive tests, SLA samples may be loaded within a range without deformation accumulation. SLA micro-deformation pattern was very similar to that of native trabecular bone, as supported by DVC with full-field strain distribution within the sample in the elastic and plastic regime. In the future, the produced scaffold can be used to set up an in vitro cell growth experiment with mechanical stimulation to study cell proliferation in response to different mechanical stimulation parameters. 

## Figures and Tables

**Table 1:** Compressive parameter for trabecular bone and SLA scaffold. Values are reported as mean and standard deviation for the six measured samples.

	E (MPa)	σ_y_ (MPa)	σ_ult_ (MPa)
Trabecular bone	336 ± 186	4.0 ± 1.2	4.7 ± 1.3
SLA sample	329 ± 48	7.9 ± 1.4	7.9 ± 1.4

## Data Availability

The data presented in this study is available on request from the corresponding author.
